# Medical News

**Published:** 1856-01

**Authors:** 


### THE MEDICAL EXAMINER.

PHILADELPHIA, JANUARY, 1856.

### MEDICAL NEWS.

THE CHILDREN'S HOSPITAL OF PHILADELPHIA.—We are pleased to notice the establishment of a hospital for sick children in our city, and under circumstances which augur well for its future success. It may seem strange to the medical profession in this country, but it is, we believe, not the less true, that this is, with one exception, the only effort that has yet been made in the United States to found an institution of this nature. The exceptional experiment, above alluded to, originated some years since with the late Amos Lawrence, Esq., of Boston, and if our memory serves us right, miscarried, or more properly speaking, was abandoned in consequence of an impression on the part of its benevolent founder, that the advantages accruing to the class for whose benefit it was designed, did not justify the large outlay required for its support. His hospital continued open for a few months, and was well filled with patients during the latter part of its career. Its failure was therefore more apparent than real, and resulted mainly from a want of previous knowledge on the part of Mr. L., of the difficulties which necessarily beset the infancy of these institutions, difficulties which disappear with increased experience and more enlarged accommodation.

The successful carrying out of an undertaking of this nature, requires unwearying kindness and patience on the part of those having charge of the sick, and a steady perseverance, in the teeth of much opposition from the prejudices of parents, who at first are unwilling to entrust their offspring to the care of strangers; but that this objection often so strongly urged, can be triumphantly met, is proved by the complete success of kindred institutions in Europe and Great Britain, where in some of the larger hospitals, as that of the "Hopitàl des Enfants," at Paris, 600 children can be accommodated.

The hospital located in Blight st., third door north of Lombard st., has been opened for a little over a fortnight. Its capacity is for 14 patients only, and these are to be received between the ages of 2 and 8 years. No contagious disorders for the present are to be taken into the house, but we understand that this very objectionable feature will be done away with in a few months and special accommodation provided for this class of patients. The ward arrangements are principally based on those of the Pennsylvania Hospital, with such modifications as are required for the peculiar wants of children.

We annex below a list of the Managers and Medical officers of the Hospital:—

| | | |
|---|---|---|
| George B. Wood, M. D.<br>M. D. Lewis,<br>Richard Wood,<br>Wm. R. Lejee,<br>John P. White,<br>Adolph Borie, | *Managers.* | George Norris, M. D.<br>Benjamin Gerhard,<br>Heyward Drayton,<br>John H. Atwood,<br>Morton P. Henry,<br>Alexander Henry. |

| | | | |
|---|---|---|---|
| William E. Pepper,<br>John F. Meigs. | *Consulting Physicians.* | T. Hewson Bache,<br>F. W. Lewis,<br>R. A. F. Penrose. | *Attending Physicians.* |

---

Coroners' Inquests.—At the last Annual Meeting of the American Association, held in the city of Philadelphia, the undersigned was appointed Chairman of a Committee, with Doctors D. Francis Condie, of Pennsylvania, and Grafton Tyler, of the District of Columbia, to report to the Association, what "measures should be adopted to remedy the evils existing in the present methods of holding 'coroners' inquests.'"

It is deemed very desirable and necessary, to obtain any data, such as the regulations in the various States and Territories of the United States, and also of foreign governments, relative to holding inquests, the tenure and qualifications of the office of coroner, the fees paid to medical experts, the various forms of procedure, and all other matter appertaining to this important subject.

Any facts, therefore, or cases of flagrant abuse or incompetence, coming under the observation of any member of the profession, or any suggestions, will be most thankfully received and duly acknowledged.

I am, respectfully, your obedient servant,

A. J. Semmes, M. D.
Chairman of Committee on Coroners' Inquests.
Washington, D. C.

---

The Stethoscope, a Virginian Medical Journal, has been discontinued, having been incorporated into the Virginia Medical and Surgical Journal.

St. Andrew's Society, Tribute of Respect to J. K. Mitchell, M. D.—At a meeting of this Society, held at the United States Hotel, on the 31st of October last, Dr. J. K. Mitchell, preparatory to the annual election of officers for 1855–6, called the attention of the Society to the declination of the Presidency, which he had offered a year before, and to the condition upon which he agreed to act as President for the year 1854–5, to wit, that at the present election his name should not be considered as before the Society for office. He stated that he was unwilling to hold any office, to the duties of which he did not give an active, personal and conscientious devotion and discharge; that the duties of the Presidency of St. Andrew's, thus attended to, were, so far onerous, as to interfere with his medical labors and Professorship. He asked therefore, as a favor to himself, to be allowed to decline this dignified situation which the Society had conferred upon him at the decease of the late President, Dr. Nathaniel Chapman.

The matter being on motion referred to a committee consisting of Messrs. John Rea, Thomas Dunlap, George Young and J. W. Wallace, to report upon, the following resolutions were reported, and on a subsequent evening unanimously adopted:—

*Resolved*, That the brethren of St. Andrew's Society, receive with sincere regret information from their President, Dr. J. K. Mitchell, of his wish to decline the chief office of the Society, which he has filled for some years past to such eminent satisfaction of the Society, and so much to its advantage in increase of numbers, harmony and general welfare.

*Resolved*, That Dr. Mitchell, having been prevailed on to accept the Presidency of the Society in 1854 only on condition, which he then expressed, that he might retire from it in 1855, and now urging the extent of his medical duties as a cause for retirement, that his resignation be accepted.

*Resolved*, That the affectionate wishes of the members of this brotherhood be offered to Dr. Mitchell, for a long continuance of health, and of his present eminent professional usefulness, both in his large circle of medical practice, and in the dignified station from which his medical teachings are received with interest and instruction, by every part of our extensive land.

*Resolved*, That these resolutions be published in the papers of Philadelphia, and in the Medical Examiner, and that a copy, attested by the newly-elected President and the Secretary, be transmitted to Dr. Mitchell.

Thomas Dunlap, *President.*
George Young, *Secretary.*

*United States Hotel, Nov. 21st*, 1855.

The Use of Chloroform in Edinburgh.—Professor Simpson states, that during the last six or seven years, few operations have been performed in Edinburgh, either in hospital or private practice, without the patient being previously anæsthetised with chloroform. During that period one death has occurred in the city, among the many thousands who have been subjected to the use of chloroform. But during the same six or seven years, among the comparatively few operated upon there without chloroform, three or four deaths have taken place, either during or immediately after the surgical operation. This statement, from such a source, is of great value.—*Medical Times.*

---

Dr. Southwood Smith has been giving a very important series of lectures in Edinburgh, on the subject of epidemics. Dr. Smith dwelt particularly, in his introductory lecture, on the fact that all epidemic diseases—the plague, black-death, sweating-sickness, cholera, influenza, &c., were fevers. Cholera was usually preceded, he stated, by influenza. In cholera, if the patient could be saved three days, the fever and other symptoms were curable. Dr. Southwood Smith seemed to say that very active animal and epidemic poisons were generated by over-crowding of human beings, and when to this were added deficient electricity in the atmosphere, unusual prevalence of mist, haze, or fog, stillness of the air, and augmented barometric pressure, then we had an epidemic constitution of things, and would have most probably cholera.—*Dublin Med. Press.*

---

Royal Hospital for Incurables.—This establishment is progressing very favorably; this week eight candidates have been elected to the institution in addition to the twenty-two previously in the asylum. The annual meeting was presided over by Mr. Alderman Wire, who made the following judicious observations on the undertaking. He said "that the experiment had been very successful, and when they recollected the circumstances under which it arose, and that it was designed to alleviate the sorrows of those for whom no other institution was provided, they would see the absolute necessity of a speedy, extensive and generous effort in its behalf. Its funds ought to be at least three times what they at present were. We had splendid hospitals, and various institutions for different forms of human suffering; but, as this was the only asylum for those who were considered incurable, he thought it had a very strong claim upon their sympathy and support. Hundreds of cases were dismissed from our great hospitals as being incurable, and they were but too frequently persons belonging to that class of society without the pecuniary means of alleviating their sufferings. Their maladies were such as medicine could not cure; but kindness and comfort would very much ameliorate their condition, though they could not effect a complete cure. Such was the class of persons for whose benefit this hospital was established, on account of which they now appealed to the benevolent public for pecuniary aid. The institution had gone on very vigorously hitherto, and he hoped that they would support it by every means in their power. There were no theological or political distinctions. The medical officers, and almost every other person, rendered gratuitous services. The entire salaries were but £91 8*s*. 8*d*. All the subscriptions, therefore, were devoted to the objects of the charity, instead of being largely absorbed in the salaries of officials. The income ought to be increased at least threefold, and he hoped it speedily would."—*London Lancet.*

[There is great need of such an Institution here, and, in fact, in each of our large cities. No express provision exists, that we are aware of, for such cases, in any part of the United States.—Ed. Ex.]

---

Anæsthetics in the Austrian Army.—A circular has just been issued, ordering that in future, the Army Medical officers shall always employ, for the purpose of inducing anæsthesia, a mixture consisting of one part chloroform and nine parts ether, this being the proportion long employed by Dr. Weiger, a Vienna Dentist.

---

Hospital for Consumption, London.—Since the opening of this Institution in 1846, 3,656 in-patients, and 29,265 out-patients have been treated.

---

Surgeon O'Callaghan of the 62d Foot.—The *London Gazette* of the 16th instant contains the following gratifying letter, dated Camp Sebastopol, October 22nd, from Major Daubeney, relative to the important services rendered by Staff Assistant Surgeon O'Callaghan, of the 62nd Regiment: "Sir,—In bringing to the notice of Major-General Windham, C. B., the names of the officers and men of the 62nd Regiment, who distinguished themselves at the assault of the Redan, on the 8th of September last, I omitted to mention the name of Staff Assistant-Surgeon O'Callaghan, who is attached to the 62nd Regiment. His attention to the wounded was not confined to men of his own regiment on that day, but was extended to officers and men of all regiments who happened to be brought past him; he accompanied the regiment as far as the fifth parallel, and volunteered to remain behind after the regiment was ordered back to camp, to assist in attending to, and bringing in, the wounded from the front at dusk. Many officers have spoken in high terms of his conduct and exertions in behalf of the wounded on that day, requesting that his services may be brought to the notice of the Commander-in-Chief.

---

Adulteration of Food.—A correspondent of the *Times* says he avoided green pickles, fearing copper, but what was his surprise to find copper in tomata sauce. He sends the *Times* the label of the bottle, which says, "all articles in the Italian line of the first quality." He *naïvely* adds, the Italian line here spoken of must be that of Lucretia Borgia and Co. Another writer in the *Times* says, "the coloring matter of Cheshire cheeses alone costs £2250 per annum; this amount being thrown away on a mixture of turmeric, potash, soft soap, and train oil, with annatto."

---

Obituary.—We have heard with sincere regret, of the recent death of Dr. J. F. Peebles, of Petersburg, one of the editors of the Virginia Medical and Surgical Journal.

---

Correction.—In Prof. Hamilton's Report on "Deformities after Fractures," in the Transactions of the American Medical Association, on page 436, 19th line from the top, the word "able," should read "unable." The publishing committee are not responsible for the error.

